# Genome Sequence-Guided Finding of Lucensomycin Production by *Streptomyces achromogenes* Subsp. *streptozoticus* NBRC14001

**DOI:** 10.3390/microorganisms10010037

**Published:** 2021-12-26

**Authors:** Sho Nishimura, Kazune Nakamura, Miyako Yamamoto, Daichi Morita, Teruo Kuroda, Takanori Kumagai

**Affiliations:** 1 School of Pharmaceutical Sciences, Department of Microbiology, Hiroshima University, Kasumi 1-2-3, Minami-ku, Hiroshima 734-8553, Japan; b161418@hiroshima-u.ac.jp; 2 Graduate School of Biomedical and Health Sciences, Department of Microbiology, Hiroshima University, Kasumi 1-2-3, Minami-ku, Hiroshima 734-8553, Japan; m213778@hiroshima-u.ac.jp (K.N.); b184843@hiroshima-u.ac.jp (M.Y.); dmorita@hiroshima-u.ac.jp (D.M.); tkuroda@hiroshima-u.ac.jp (T.K.)

**Keywords:** genome-guided approach, lucensomycin, polyene macrolide, antifungal compound, *Streptomyces*

## Abstract

Information on microbial genome sequences is a powerful resource for accessing natural products with significant activities. We herein report the unveiling of lucensomycin production by *Streptomyces achromogenes* subsp. *streptozoticus* NBRC14001 based on the genome sequence of the strain. The genome sequence of strain NBRC14001 revealed the presence of a type I polyketide synthase gene cluster with similarities to a biosynthetic gene cluster for natamycin, which is a polyene macrolide antibiotic that exhibits antifungal activity. Therefore, we investigated whether strain NBRC14001 produces antifungal compound(s) and revealed that an extract from the strain inhibited the growth of *Candida albicans*. A HPLC analysis of a purified compound exhibiting antifungal activity against *C. albicans* showed that the compound differed from natamycin. Based on HR-ESI-MS spectrometry and a PubChem database search, the compound was predicted to be lucensomycin, which is a tetraene macrolide antibiotic, and this prediction was supported by the results of a MS/MS analysis. Furthermore, the type I polyketide synthase gene cluster in strain NBRC14001 corresponded well to lucesomycin biosynthetic gene cluster (*lcm*) in *S. cyanogenus*, which was very recently reported. Therefore, we concluded that the antifungal compound produced by strain NBRC14001 is lucensomycin.

## 1. Introduction

Actinomycetes are industrially important microorganisms from which large numbers of secondary metabolites have been isolated, and several are used as beneficial agents that exhibit anti-bacterial, anti-cancer, anthelmintic, and immunosuppressive activities [1]. Until the end of the 20th century, access to the compounds produced by actinomycetes was mainly based on the following schemes: the initial isolation of actinomycete strains from natural sources, such as soil samples, followed by their cultivation in a liquid medium or on a solid medium, and the separation and isolation of the desired compounds from the medium based on their activities, such as anti-bacterial activity [2]. However, in the 21st century, the way of access to secondary metabolites of actinomycetes has markedly changed because large numbers of genome sequences from actinomycetes have been accumulated due to advances in sequencing technology. The discovery of proteins involved in the biosynthesis of secondary metabolites may be now achieved by next-generation sequencing chemistry. Secondary metabolite biosynthetic gene clusters (smBGCs) in actinomycete strains are extracted by in silico analyses using programs such as antiSMASH [3]. Based on the information obtained on smBGCs, secondary metabolites are unveiled using appropriate procedures, such as biotechnological approaches including heterologous expression and promoter engineering [4,5,6,7].

*Streptomyces achromogenes* subsp. *streptozoticus* NBRC14001, which was used in the present study, is a producer of streptozotocin (STZ) [8,9,10]. STZ is an antibiotic that inhibits the growth of both Gram-positive and Gram-negative bacteria [10]. STZ has recently been used to treat pancreatic and gastroenteric neuroendocrine tumors [11]. Although strain NBRC14001 produces STZ, no other secondary metabolites produced by this strain have been reported until now.

In the present study, to identify secondary metabolites other than STZ produced by strain NBRC14001, we elucidated the genome sequence of the strain and detected a Type I polyketide synthase (PKS) gene cluster resembling a natamycin (NTM) BGC [12] in its genome using an antiSMASH analysis. Based on this information, we herein revealed that *S. achromogenes* subsp. *streptozoticus* NBRC14001 produces lucensomycin (LCM), a tetraene macrolide antibiotic that exhibits antifungal activity [13]. LCM is an important antibiotic because it is useful for postharvest disease control against gray mold on grapes [14].

## 2. Materials and Methods

### 2.1. Strains and Culture Conditions

*S. achromogenes* subsp. *streptozoticus* NBRC14001 was obtained from the Biological Resource Center, NITE (National Institute of Technology and Evaluation), Japan, and maintained on a solid medium (1% maltose, 1% yeast extract, 0.1% beef extract, 0.2% tryptone, and 2% agar, pH 7.2) at 28 °C. To isolate its genomic DNA, strain NBRC14001 was grown in 50 mL of a liquid medium (1% glucose, 0.5% glycerol, and 0.4% yeast extract, pH 7.0) at 28 °C and 200 rpm for 48 h. Regarding antibiotic production, strain NBRC14001 was cultivated in 100 mL of a pre-culture medium (2.5% glucose, 2.5% soybean flour, 0.2% CaCO_3_, and 5 mM MgCl_2_, pH 7.2) at 28 °C and 200 rpm for 48 h, and 1 mL of the pre-culture broth was then inoculated into 100 mL of the production medium (0.3% glucose, 1.5% soluble starch, 4% corn flour, 0.3% tryptone, 0.2% CaCO_3_, and 5 mM MgCl_2_, pH 7.2) and cultured at 28 °C and 200 rpm for 6 days.

In bioassays, *Candida albicans* YU1200 was grown on a solid medium (1% glucose, 1% hypolypepton, 0.5% beef extract, 0.25% NaCl, and 1.5% agar, pH 7.2) at 28 °C for 24 h.

### 2.2. Genome Sequencing and In Silico Analysis

The genomic DNA was isolated by a previously described method [15]. Genome sequencing was performed using the PacBio RSII sequencer (Pacific Biosciences, Menlo Park, CA, USA) at Macrogen Japan Corp. (Tokyo, Japan). The subreads obtained by sequencing were assembled using the HGAP program [16], and annotation of the resulting genome sequence was conducted by the Prokka ver. 1.12 program [17]. smBGCs in the genome of strain NBRC14001 were analyzed using the antiSMASH ver. 6.0 program [3]. Predictions of the molecular formula and compound were performed by the Kazusa MFSearcher [18] website (http://webs2.kazusa.or.jp/mfsearcher accessed on 10 August 2021) and PubChem [19] website (https://pubchem.ncbi.nlm.nih.gov accessed on 10 August 2021), respectively. Similarity analyses between each open reading frame (ORF) in the type I PKS cluster in strain NBRC14001 and the corresponding ORF in LCM BGC in *S. cyanogenus* S136 [20] were conducted using the Blastp program [21] in align two or more sequences mode. In addition, similarity analyses of ORFs in the type I PKS cluster in strain NBRC14001 against non-redundant protein sequences were also conducted by the Blastp program (E-value cutoff was not set).

The nucleotide sequences of the type I PKS gene cluster, which is responsible for LCM production in strain NBRC14001, have been deposited in DNA Data Bank of Japan (DDBJ) (https://www.ddbj.nig.ac.jp/ 28 October 2021) under the accession number LC656361.

### 2.3. Pilot Antifungal Activity Test

To investigate whether strain NBRC14001 produces antifungal compound(s), the strain was cultured in a 100 mL of production medium. After cultivation, culture supernatant and mycelia were separated by centrifugation. The culture supernatant was extracted with an equal volume of *n*-butanol, and the solvent of the organic layer was evaporated under reduced pressure. The resulting residue was dissolved in a small volume of methanol and used as a sample for bioassay using *C. albicans* as a test organism. The mycelia were extracted with 100 mL of acetone and the solvent was evaporated under reduced pressure. The resulting residue was dissolved in a small volume of methanol and used as a sample for bioassay using *C. albicans* as a test organism.

### 2.4. Isolation of an Antifungal Compound

An antifungal compound was isolated from the culture broth of strain NBRC14001 by chromatography. The antifungal activity of each fraction obtained by chromatography was assessed as follows: Each fraction was dried by evaporation and freeze drying. The resulting residue was dissolved in a small volume of methanol and used as a sample for bioassay using *C. albicans* as a test organism. An antifungal compound was isolated from 4 L of culture broth. Briefly, 4 L of culture broth was filtrated under reduced pressure, and the resulting culture supernatant was extracted with a double volume of *n*-butanol once. The solvent of the organic layer was evaporated under reduced pressure, and the residue was dissolved in chloroform and applied to a silica gel (Nacalai Tesque, Kyoto, Japan) column (3.0 × 30 cm). Elution was successively performed with 200 mL of chloroform: methanol = 1:0, 4:1, 3:2, 2:3, 1:4, and 0:1. The solvent of the active fractions was evaporated under reduced pressure, and the resulting residue was dissolved in 50% acetonitrile in water and applied to COSMOSIL 75C_18_-OPN (Nacalai Tesque, Kyoto, Japan) column (3.0 × 30 cm). Elution was isocratically conducted with the same solvent (50% acetonitrile in water). Active fractions were dried by evaporation and freeze drying, and then separated by preparative HPLC with the ODS-AQ column (20 × 250 mm, YMC, Kyoto, Japan) using 60% methanol in water (solvent A) and 70% methanol in water (solvent B) as solvents. Elution was performed with A100% to B100% for 1 h, and then with B100% for 1 h at a flow rate of 3.0 mL/min.

### 2.5. HPLC Analysis of a Purified Antifungal Compound

An active peak obtained by preparative HPLC was analyzed by HPLC with the COSMOSIL 5C_18_-PAQ column (4.6 × 250 mm, Nacalai Tesque, Kyoto, Japan) using 60% methanol in water as a mobile phase at a flow rate of 1.0 mL/min. NTM was purchased from Toronto Research Chemicals Inc. (Toronto, ON, Canada) and used for comparisons with the active compound from strain NBRC14001.

### 2.6. ESI-MS and MS/MS Spectrometry

ESI-MS spectrum and HR-ESI-MS analyses were performed with the Exactive MS spectrometer (Thermo Fisher Scientific, Waltham, MA, USA). A MS/MS analysis was conducted using the LTQ-Orbitrap XL MS spectrometer (Thermo Fisher Scientific, Waltham, MA, USA). All MS analyses were outsourced to the Instrumental Analysis Division, Global Facility Center, Creative Research Institution, Hokkaido University, Japan.

## 3. Results

### 3.1. The Genome-Guided Approach Revealed That Strain NBRC14001 Produces Compound(s) That Exhibit Antifungal Activity

After assembly and annotation, we elucidated the genome sequence of strain NBRC14001 consisting of 8,897,387 bp by PacBio RSII sequencing (genome sequence has not yet been deposited in public database). The antiSMASH analysis [3] of the genome sequence revealed that a type I PKS gene cluster (*sac_7029* ~ *sac_7047*), which exhibits moderately high similarity to a NTM BGC [12], is present in the genome. Fourteen out of 21 genes in NTM BGC exhibit similarities to genes involved in type I PKS cluster of strain NBRC14001 (that is, 67% of genes in NTM BGC show similarities to genes in the type I PKS cluster of NBRC14001). 

**Figure 1:**
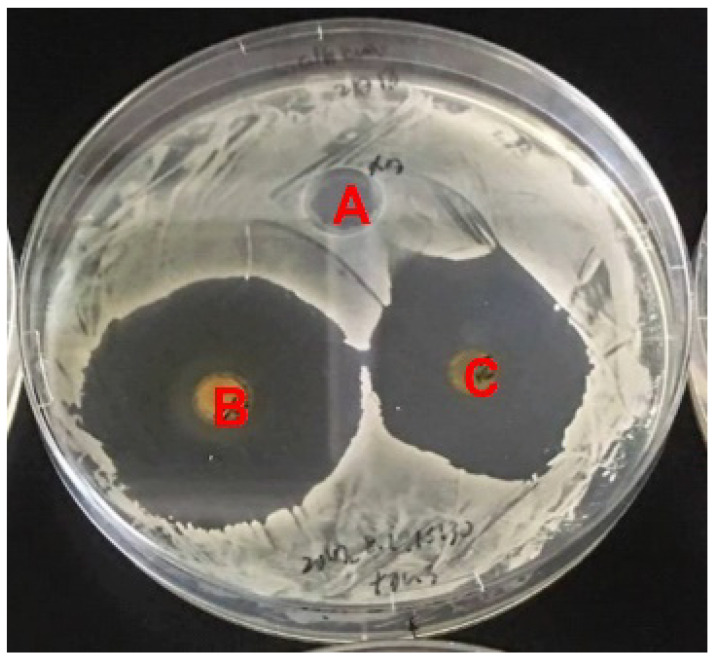
Antifungal activity of an extract from *S. achromogenes* subsp. *streptozoticus* NBRC14001. *Candida albicans* was used as a test organism. A, methanol (control); B, *n*-butanol extract from the culture supernatant; C, acetone extract from mycelia.

NTM is a tetraene macrolide antibiotic that is produced by *S. natalensis* and exhibits antifungal activity [13,22]. Therefore, we hypothesized that strain NBRC14001 may produce compound(s) with antifungal activity. To test this hypothesis, we performed a bioassay using *C. albicans* as a test organism. As shown in Figure 1, the extract from the culture supernatant (*n*-butanol extract) (B) and that from mycelia (acetone extract) (C) inhibited the growth of *C. albicans*, which demonstrated that strain NBRC14001 produces antifungal compound(s).

### 3.2. The Active Compound Exhibiting Antifungal Activity Is Different from NTM

**Figure 2:**
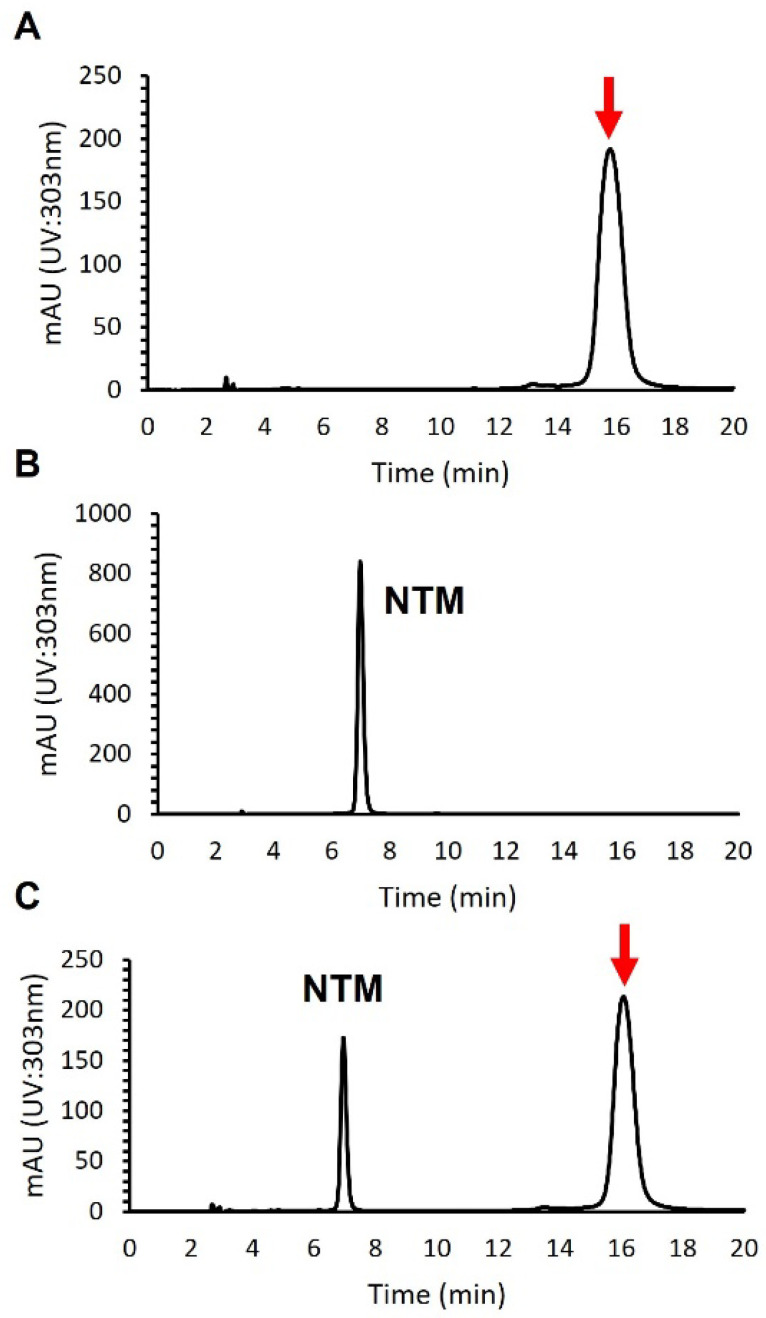
HPLC analysis of an antifungal compound isolated from *S. achromogenes* subsp. *streptozoticus* NBRC14001: (**A**) an antifungal compound from NBRC14001; (**B**) NTM; (**C**) co-injection of an antifungal compound from NBRC14001 and NTM. A red arrow indicates the peak of isolated compound.

An antifungal compound was isolated from 4 L of culture broth by normal phase open column chromatography using a silica gel, reversed-phase open column chromatography with an ODS filler, and preparative HPLC using an ODS column (see Section 2). To distinguish the isolated compound from NTM, we performed a HPLC analysis. As shown in Figure 2, the retention time of the compound appeared to differ from that of NTM, which demonstrated that the compound isolated was not NTM.

### 3.3. Structural Analysis of the Antifungal Compound

**Figure 3:**
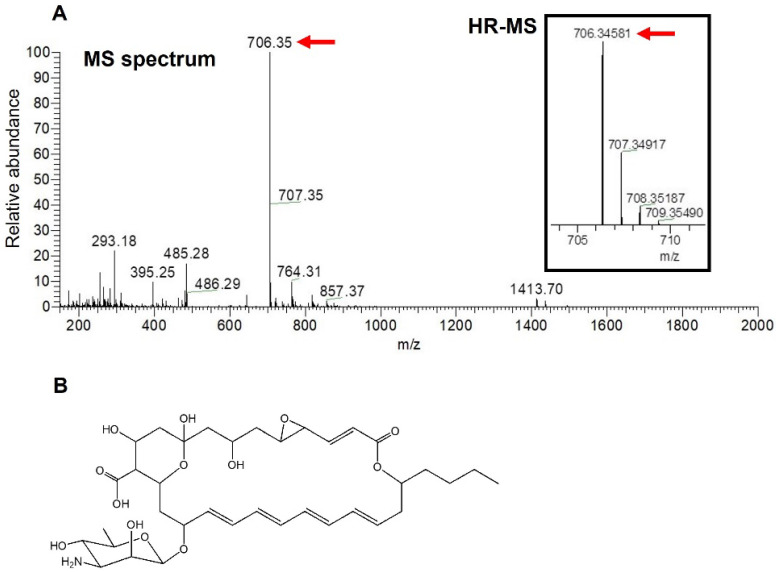
MS analyses of an antifungal compound isolated from *S. achromogenes* subsp. *streptozoticus* NBRC14001: (**A**) MS spectrum and HR-MS analyses; (**B**) structure of LCM. A red arrow indicates the *m*/*z* of isolated compound.

Since structure determination by the nuclear magnetic resonance (NMR) analysis of the antifungal compound produced by strain NBRC14001 was hampered by the very low yield of the compound, we attempted to elucidate its structure using MS analyses. An ESI-MS spectrum analysis was initially performed, and a peak having *m*/*z* = 706.35 [M − H]^−^ was obtained (Figure 3A). A HR-ESI-MS analysis was conducted based on this peak. The results obtained revealed that the observed *m*/*z* of the peak was 706.3458 [M − H]^−^ (Figure 3A, in parenthesis). In consideration of the observed *m*/*z* and structures of tetraene macrolide antibiotics isolated to date, the molecular formula of the antifungal compound was predicted to be C_36_H_53_NO_13_ (*m*/*z* = 706.3444 [M − H]^−^, calculated, mass tolerance 5 ppm). A search for the molecular formula (C_36_H_53_NO_13_) in the PubChem database [19] indicated that the antifungal compound isolated was LCM (Figure 3B), a tetraene macrolide isolated from *S. lucensis* with antifungal activity [13,22].

**Figure 4:**
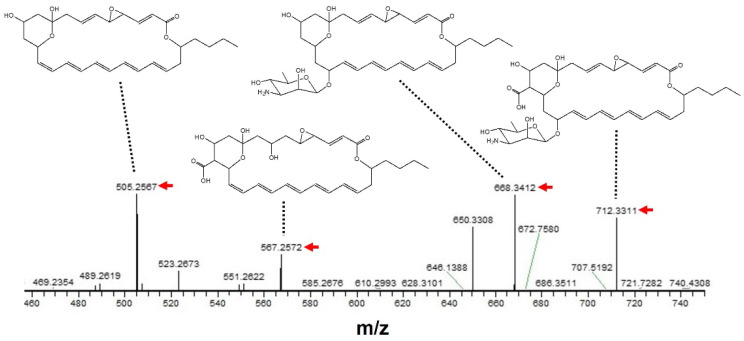
MS/MS analysis of an antifungal compound isolated from *S. achromogenes* subsp. *streptozoticus* NBRC14001. A peak having *m*/*z* = 730.3416 [M + Na]^+^ was used as a precursor ion. A red arrow indicates the *m*/*z* of each fragment peak.

To further support the above prediction, a MS/MS analysis was performed based on the peak having *m*/*z* = 730.3416 [M + Na]^+^ as a precursor ion. As shown in Figure 4, the *m*/*z* of each fragment peak observed was considered to be a sodium adduct of each LCM-derived structure, strongly supporting the compound isolated in the present study being LCM.

### 3.4. Verification of LCM Production by Comparisons of BGCs

**Figure 5:**
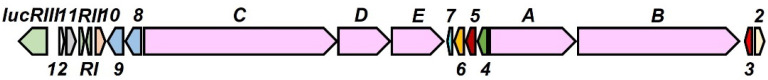
Gene organization of the type I PKS cluster (*luc*) in *S. achromogenes* subsp. *streptozoticus* NBRC14001.

A gene cluster (named *lcm*) responsible for the production of LCM in *S. cyanogenus* S136 was very recently reported [20]. Therefore, ORFs (Sac_7029 ~ Sac_7047) in the type I PKS cluster of strain NBRC14001 were compared with those in the LCM BGC of *S. cyanogenus* using the Blastp program [21] in two or more sequences mode. All Lcm homologs were revealed to be conserved in the type I PKS cluster of NBRC14001. This result further supported the antifungal compound produced by strain NBRC14001 being LCM. Therefore, gene names in the type I PKS cluster (*sac_7029* ~ *sac_7047*) were renamed as shown in Figure 5, and ORFs involved in the cluster are summarized in Table 1. Similarity analyses of the ORFs involved in *luc* cluster against non-redundant protein sequences were also performed by the Blastp program (E-value cutoff was not set) and the results are summarized in Table S1.

We also investigated domain structures in each module of PKSs (LucA ~ LucE) and compared them with those of PKSs in *S. cyanogenus* S136. The results obtained revealed that the domain structures of strain NBRC14001 corresponded perfectly with those of *S. cyanogenus* (Table 2). Based on these results, we concluded that the antifungal compound produced by the strain NBRC14001 is LCM.

## 4. Discussion

In the present study, based on genome sequence information, we identified a Type I PKS cluster that shows similarity to NTM BGC using an antiSMASH analysis [3]. Since the LCM BGC (*lcm*) of *S. cyanogenus* S136 has very recently been reported [20], information on the cluster may not yet be registered, even in the latest version of the antiSMASH program. Due to the potential absence of information, the analysis using the latest version of the antiSMASH program may not have shown that the type I PKS cluster in NBRC14001 resembles the *lcm* cluster. Furthermore, although we examined the ORFs of the type I PKS cluster in NBRC14001 directly using the Blastp program [21], it was difficult to reach the conclusion that the type I PKS cluster in NBRC14001 is closely related to the *lcm* cluster because large numbers of proteins were hit by the Blastp analysis. Therefore, we noted that our cluster (*luc*) corresponded well to the *lcm* cluster at the time that the antifungal compound we isolated was revealed to potentially be LCM.

Based on Table 1 and Table S1, we propose function of each ORF in the *luc* cluster in the biosynthesis of LCM. The type I PKSs, LucA~LucE, are most likely to catalyze the formation of LCM aglycon [20]. The putative type II thioesterase, Luc3, may play a role in proofreading during the formation of LCM aglycon [23]. The putative cytochrome P450s, Luc6 and Luc10, are probably involved in the post-PKS modifications. Luc6 is considered to catalyze carboxylation at C-12 position of aglycon, whereas Luc10 is a candidate for the catalysis of epoxidation at C4-C5 positions [20,24]. The putative ferredoxin Luc7 may function as an electron donor for Luc6 and/or Luc10. Luc2, Luc4, and Luc5 are also likely to play roles in the post-PKS modifications. The putative dehydratase Luc2 and aminotransferase Luc5 are probably involved in the formation of GDP-mycosamine from GDP-mannose, whereas the putative glycosyltransferase Luc4 is considered to catalyze the transfer of mycosamine to LCM aglycon [20]. There are three putative transcriptional regulators present in the *luc* cluster (LucRI, RII, and RIII). As in the case of pathway specific regulators present in pimaricin (equal to NTM) and nystatin BGCs [25,26,27], they might control the biosynthesis of LCM in strain NBRC14001. The putative ABC transporter, Luc8 and Luc9, are probably involved in the transport of LCM from inside cells to outside [20]. The function of remaining two ORFs (Luc11 and Luc12) in the biosynthesis of LCM is unclear. We intend to elucidate function of each ORF in the LCM biosynthesis by the gene disruption experiments in future works.

A previous study reported that LCM production in *S. cyanogenus* S136 was unveiled by introducing a heterologous *adpA* gene into the strain [20]. The AdpA protein encoded by *adpA* is a AraC family transcriptional regulator that globally regulates cellular functions, such as morphological differentiation and secondary metabolism, in actinomycetes [28]. Since AdpA (SCY4743) in *S. cyanogenus* S136 is non-functional, the expression of heterologous *adpA* was necessary for LCM production in the strain [20]. In contrast to the case in *S. cyanogenus* S136, strain NBRC14001 produced LCM without expressing the *adpA* gene, and a gene encoding *adpA* was absent in the genome of NBRC14001; however, several genes encoding putative AraC family transcriptional regulators were present (our unpublished data). The regulation of LCM production in each strain may differ from each other. Therefore, further studies are warranted to clarify the effects of heterologous *adpA* expression on LCM production in NBRC14001. 

## 5. Conclusions

In the present study, based on a genome-guided approach, the antifungal compound isolated from NBRC14001 was revealed to be the known tetraene macrolide antifungal antibiotic, LCM, and the BGC for LCM in *S. cyanogenus* S136 has already been reported. However, the present study showed for the first time that the strain produces a secondary metabolite other than STZ. As a number of novel compounds have been recently identified by the genome-guided approach [4,5,6,7], this approach may become a powerful tool for investigating novel compounds in microorganisms.

## Figures and Tables

**Table 1:** ORFs involved in the Type I PKS cluster (*luc*) in *S. achromogenes* subsp. *streptozoticus* NBRC14001.

ORF	aa	Putative Protein	Lcm Homolog	Identity /Positivity (%)
Luc2	344	GDP-D-mannose 4,6-dehydratase	Lcm2	94/97
Luc3	257	Type II thioesterase	Lcm3	86/91
LucA	3035	Type I polyketide synthase (module 0–1)	LcmA	90/93
LucB	6457	Type I polyketide synthase (module 2–5)	LcmB	87/91
Luc4	469	GDP-mycosamine glycosyltransferase	Lcm4	82/90
Luc5	352	GDP-3-keto-6-deoxy-D-mannose C-3 aminotransferase	Lcm5	73/83
Luc6	392	Cytochrome P450 monooxygenase	Lcm6	92/97
Luc7	64	Ferredoxin	Lcm7	88/93
LucC	9249	Type I polyketide synthase (module 6–11)	LcmC	91/94
LucD	1789	Type I polyketide synthase (module 12)	LcmD	90/93
LucE	1995	Type I polyketide synthase (module 13)	LcmE	91/94
Luc8	578	ABC transporter	Lcm8	90/93
Luc9	625	ABC transporter	Lcm9	89/93
Luc10	386	Cytochrome P450 monooxygenase	Lcm10	90/94
LucRI	204	HTH-LuxR domain-containing transcriptional regulator	LcmRI	83/90
LucRII	230	HTH-LuxR domain-containing transcriptional regulator	LcmRII	83/89
Luc11	548	Cholesterol oxidase	Lcm11	91/95
Luc12	100	Hypothetical protein		
LucRIII	1188	Transcriptional regulator	LcmRIII	82/86

**Table 2:** Domain structures in each module of PKSs from *luc* cluster in *S. achromogenes* subsp. *streptozoticus* NBRC14001 and *lcm* cluster in *S. cyanogenus* S136.

Cluster	PKS	Module	Domain Structure
*luc* cluster			
	LucA	0	KS-AT-ACP
		1	KS-AT-DH-ER-KR-ACP
	LucB	2	KS-AT-KR-ACP
		3	KS-AT-DH-KR-ACP
		4	KS-AT-DH-KR-ACP
		5	KS-AT-DH-KR-ACP
	LucC	6	KS-AT-DH-KR-ACP
		7	KS-AT-KR-ACP
		8	KS-AT-KR-ACP
		9	KS-AT-KR-ACP
		10	KS-AT-ACP
		11	KS-AT-KR-ACP
	LucD	12	KS-AT-DH-KR-ACP
	LucE	13	KS-AT-DH-KR-ACP-TE
*lcm* cluster			
	LcmA	0	KS-AT-ACP
		1	KS-AT-DH-ER-KR-ACP
	LcmB	2	KS-AT-KR-ACP
		3	KS-AT-DH-KR-ACP
		4	KS-AT-DH-KR-ACP
		5	KS-AT-DH-KR-ACP
	LcmC	6	KS-AT-DH-KR-ACP
		7	KS-AT-KR-ACP
		8	KS-AT-KR-ACP
		9	KS-AT-KR-ACP
		10	KS-AT-ACP
		11	KS-AT-KR-ACP
	LcmD	12	KS-AT-DH-KR-ACP
	LcmE	13	KS-AT-DH-KR-ACP-TE

Abbreviations: ACP, acyl carrier protein; AT, acyltransferase; DH, dehydratase; ER, enoyl reductase; KR, ketoreductase; KS, ketosynthase; TE, thioesterase.

## Data Availability

The sequences of LCM BCG have been deposited in DDBJ under the accession number LC656361.

## Supplementary Materials

The following supporting information can be downloaded at: https://www.mdpi.com/article/10.3390/microorganisms10010037/s1, Table S1: Similarity analyses of ORFs in *luc* cluster against non-redundant protein sequences.

## References

[1] Demain A.L., Sanchez S. (2009). Microbial drug discovery: 80 years of progress. J. Antibiot..

[2] Takahashi Y., Omura S. (2003). Isolation new actinomycete strains for the screening of new bioactive compounds. J. Gen. Appl. Microbiol..

[3] Blin K., Shaw S., Kloosterman A.M., Charlop-Powers Z., von Wenzel G.P., Medema M.H., Weber T. (2021). antiSMASH 6.0: Improving cluster detection and comparison capabilities. Nucl. Acids Res..

[4] Singh T.A., Passari A.K., Jajoo A., Bhasin S., Gupta V.K., Hashem A., Alqarawi A.A., Allah E.F.A. (2021). Tapping into actinobacterial genome for natural product discovery. Front. Microbiol..

[5] Kang H.-S., Kim E.-S. (2021). Recent advances in heterologous expression of natural product biosynthetic gene clusters in *Streptomyces* hosts. Curr. Opin. Biotechnol..

[6] Liu L.L., Jiang W., Lu Y. (2019). Recent advances in synthetic biology approaches to optimize production of bioactive natural products in actinobacteria. Front. Microbiol..

[7] Lee N., Hwang S., Kim J., Cho S., Palsson B., Cho B.-K. (2020). Genome mining approaches for the identification of secondary metabolite biosynthetic gene clusters in *Streptomyces*. Comput. Struct. Biotechnol. J..

[8] Vavra J.J., Deboer C., Dietz A., Hanka L.J., Sokolski W.T. (1959–1960). Streptozotocin, a new antibacterial antibiotic. Antibiot. Annu..

[9] Herr R.R., Eble T.E., Bergy M.E., Jahnke H.K. (1959–1960). Isolation and characterization of streptozotocin. Antibiot. Annu..

[10] Sokolski W.T., Vavra J.J., Hanka L.J. Assay methods and antibacterial studies on streptozotocin. Antibiot. Annu..

[11] Kiesewetter B., Raderer M. (2020). How I treat neuroendocrine tumors. ESMO Open.

[12] Aparicio J.F., Colina A.J., Ceballos E., Martin J.F. (1999). The biosynthetic gene cluster for the 26-memberd ring polyene macrolide pimaricin. A new polyketide synthase organization encoded by two subclusters separated by functionalization genes. J. Biol. Chem..

[13] Hamilton-Miller J.M.T. (1973). Chemistry and biology of the polyene macrolide antibiotics. Bacteriol. Rev..

[14] Kim J.D., Kang J.E., Kim B.S. (2020). Postharvest disease control efficacy of the polyene macrolide lucensomycin produced by *Streptomyces plumbeus* strain CA5 against gray mold on grapes. Postharvest Biol. Technol..

[15] Kieser T., Bibb M.J., Buttner M.J., Chater K.F., Hopwood D.A. (2000). Practical Streptomyces Genetics.

[16] Chin C.S., Alexander D.H., Marks P., Klammer A.A., Drake J., Heiner C., Clum A., Copeland A., Huddleston J., Eichler E.E. (2013). Nonhybrid, finished microbial genome assemblies from long-read SMRT sequencing data. Nat. Methods.

[17] Seemann T. (2014). Prokka: Rapid prokaryotic genome annotation. Bioinformatics.

[18] Sakurai N., Ara T., Kanaya S., Nakamura Y., Iijima Y., Enomoto M., Motegi T., Aoki K., Suzuki H., Shibata D. (2013). An application of a relational database system for high-throughput prediction of elemental compositions from accurate mass values. Bioinformatics.

[19] Kim S. (2021). Exploring chemical information in PubChem. Curr. Protoc..

[20] Yushchuk O., Ostash I., Mösker E., Vlasiuk I., Deneka M., Rückert C., Busche T., Fedorenko V., Kalinowski J., Süssmuth R.D. (2021). Eliciting the silent lucensomycin biosynthetic pathway in *Streptomyces cyanogenus* S136 via manipulation of the global regulatory gene *adpA*. Sci. Rep..

[21] Altschul S.F., Gish W., Miller W., Myers E.W., Lipman D.J. (1990). Basic local alignment search tool. J. Mol. Biol..

[22] Struyk A.P., Hoette I., Drost G., Waisvisz J.M., Van Eek T., Hoogerheide J.C. Pimaricin, a new antifungal antibiotic. Antibiot. Annu..

[23] Zhou Y., Meng Q., You D., Li J., Chen S., Zhou X., Zhou H., Bai L., Deng Z. (2008). Selective removal of aberrant extender units by a type II thioesterase for efficient FR-008/candicidin biosynthesis in *Streptomyces* sp. Strain FR-008. Appl. Environ. Microbiol..

[24] Mendes M.V., Antón N., Martín J.F., Aparicio J.F. (2005). Characterization of the polyene macrolide P450 epoxidase from *Streptomyces natalensis* that converts de-epoxypimaricin into pimaricin. Biochem. J..

[25] Antón N., Mendes M.V., Martín J.F., Aparicio J.F. (2004). Identification of PimR as a positive regulator of pimaricin biosynthesis in *Streptomyces natalensis*. J. Bacteriol..

[26] Antón N., Santos-Aberturas J., Mendes M.V., Guerra S.M., Martín J.F., Aparicio J.F. (2007). PimM, a PAS domain positive regulator of pimaricin biosynthesis in *Streptomyces natalensis*. Microbiology.

[27] Sekurova O.N., Brautaset T., Sletta H., Borgos S.E.F., Jacobsen Ø.M., Ellingsen T.E., Strøm A.R., Valla S., Zotchev S.B. (2004). In vivo analysis of the regulatory genes in the nystatin biosynthetic gene cluster of *Streptomyces noursei* ATCC11445 reveals their different control over antibiotic biosynthesis. J. Bacteriol..

[28] Ohnishi Y., Yamazaki H., Kato J.Y., Tomono A., Horinouchi S. (2005). AdpA, a central transcriptional regulator in the A-factor regulatory cascade that leads to morphological development and secondary metabolism in *Streptomyces griseus*. Biosci. Biotechnol. Biochem..

