# Supplementary material for: Genome Sequence-Guided Finding of Lucensomycin Production by Streptomyces achromogenes Subsp. streptozoticus NBRC14001
Source: Microorganisms. 2021 Dec 26;10(1):37. doi: 10.3390/microorganisms10010037 (PMC8781583; doi:10.3390/microorganisms10010037)
Supplement: Supplementary file 1 [file microorganisms-10-00037-s001.zip › microorganisms-1514496-supplementary.pdf]

Table S1. Similarity analyses of ORFs in *luc* cluster against non-redundant protein sequences.

ORF	aa	Best hit	Organism	Coverage (%)	E-value	Identity (%)	Accession
Luc2	344	GDP-mannose 4,6-dehydratase	*S. mutabilis*	100	0.0	97.1	WP_043386256.1
Luc3	257	Alpha/beta fold hydrolase	*S. mutabilis*	100	$1e^{-171}$	91.1	WP_043386255.1
LucA	3,035	Type I polyketide synthase	*S. viridosporus*	100	0.0	96.3	WP_081238284.1
LucB	6,457	Type I polyketide synthase	*S. cyanogenus*	100	0.0	87.4	QSE03601.1
Luc4	469	Glycosyltransferase	*S. mutabilis*	100	0.0	95.7	WP_191888611.1
Luc5	352	DegT/DnrJ/EryC1/StrS family aminotransferase	*S. mutabilis*	100	0.0	97.4	WP_191888610.1
Luc6	392	Cytochrome P450	*S. mutabilis*	100	0.0	96.9	WP_191888609.1
Luc7	64	Ferredoxin	*S. viridosporus*	100	$9e^{-38}$	100	WP_081238288.1
LucC	9,249	Type I polyketide synthase	*S. viridosporus*	100	0.0	96.4	WP_081238291.1
LucD	1,789	Type I polyketide synthase	*S. viridosporus*	100	0.0	96.3	WP_081238290.1
LucE	1,995	Type I polyketide synthase	*S. viridosporus*	100	0.0	96.4	WP_081238289.1
Luc8	578	ABC transporter ATP-binding protein/permease	*S. mutabilis*	97	0.0	95.1	WP_191888606.1
Luc9	625	ABC transporter ATP-binding protein/permease	*S. mutabilis*	100	0.0	95.0	WP_043386525.1
Luc10	386	Cytochrome P450	*S. mutabilis*	100	0.0	96.6	WP_191888604.1
LucRI	204	LuxR C-terminal-related transcriptional regulator	*S. mutabilis*	100	$1e^{-132}$	92.2	WP_191888603.1
LucRII	230	LuxR C-terminal-related transcriptional regulator	*S. viridosporus*	100	$6e^{-153}$	93.0	WP_081238295.1
Luc11	548	GMC oxidoreductase	*S. viridosporus*	100	0.0	94.9	WP_081238296.1
Luc12	100	SDR family NAD(P)-dependent oxidoreductase	*S. poonensis*	34	$1e^{-09}$	85.3	WP_229859256.1
LucRIII	1,188	AfsR/SARP family transcriptional regulator	*S. poonensis*	99	0.0	92.2	GGZ19676.1

Abbreviations used are: GMC, glucose-methanol-choline; SDR, short-chain dehydrogenases/reductases; SARP, *Streptomyces* antibiotic regulatory protein.
